# Muscle weakness and myalgia as the initial presentation of serous ovarian carcinoma: a case report

**DOI:** 10.1186/1757-2215-7-43

**Published:** 2014-04-23

**Authors:** Kyung-Jin Min, Yung-Taek Ouh, Hye-Ri Hong, Kyeong-A So, Jin Hwa Hong, Jae-Kwan Lee

**Affiliations:** 1 Department of Obstetrics and Gynecology, Korea University Medical Center, Seoul, Korea

**Keywords:** Paraneoplastic necrotizing myopathy, Ovarian carcinoma

## Abstract

**Introduction:**

Epithelial ovarian cancer (EOC) has one of the worst prognoses among gynecologic cancers. An appropriate screening method is not available for EOC, and the initial symptoms such as abdominal pain or bloating, anorexia, and urinary urgency are vague. As a result, most cases of EOC are diagnosed at an advanced stage.

**Case presentation:**

We report novel insights gained from the case of a 45-year-old, gravida 0, para 0 woman who presented to the emergency department with complaints of general weakness, fatigue, and myalgia over the previous two months. She reported progressive muscle weakness of the upper and lower extremities leading to difficulty walking. Serum muscle enzymes, such as creatine phosphokinase, were markedly elevated. No evidence of malignancy was detected upon imaging. A biopsy of the left vastus medialis muscle was performed, and the results were consistent with primary myopathy with myofibrillar disarray, suggesting paraneoplastic necrotizing myopathy. Explorative laparotomy was performed to evaluate these results, and histopathological analysis of the full specimen revealed a grade 3 ovarian serous adenocarcinoma with direct invasion to the rectum.

**Conclusions:**

Because of the lack of screening tools for EOC, any clinical findings suggesting its presence are valuable, and the possibility of EOC should be considered in unknown primary malignancies with initial complaints of muscle weakness or myalgia.

## Background

Epithelial ovarian cancer (EOC) has the one of the worst prognoses among gynecologic cancers [1,2]. No appropriate screening method is available for EOC, and the initial symptoms are vague, including abdominal pain, abdominal bloating, anorexia, and urinary urgency [3]. As a result, most cases of EOC are not diagnosed until the disease has reached an advanced stage.

Often, cancers can present as paraneoplastic syndromes, which are disorders associated with systemic cancer and are caused by mechanisms other than direct invasion or metastasis [4]. Among them, paraneoplastic necrotizing myopathy (PNM) is a rare entity characterized by a rapidly progressive, symmetric, painful, predominantly proximal muscle weakness leading to severe disability [5]. PNM is mostly associated with primary cancers of the lung, breast, and gastrointestinal tract [6-8], and until now, it has not been reported in EOC. Notably, the myositides associated with EOC are usually dermatomyositis or polymyositis [9,10], not PNM.

We report a case of a patient with initial complaints of muscle weakness and myalgia but none of the common symptoms of ovarian cancer. After a thorough workup, this patient was finally diagnosed with adenocarcinoma of the ovary with multiple metastases.

## Case presentation

A 45-year-old, gravida 0, para 0 woman presented to the emergency department with complaints of general weakness, fatigue, and myalgia over the previous two months. She described a progressive muscle weakness of the upper and lower extremities, leading to difficulty walking. The patient had no history of diabetes mellitus, alcoholism, toxin exposure, nutritional deficiency, or medication use.

**Figure 1:**
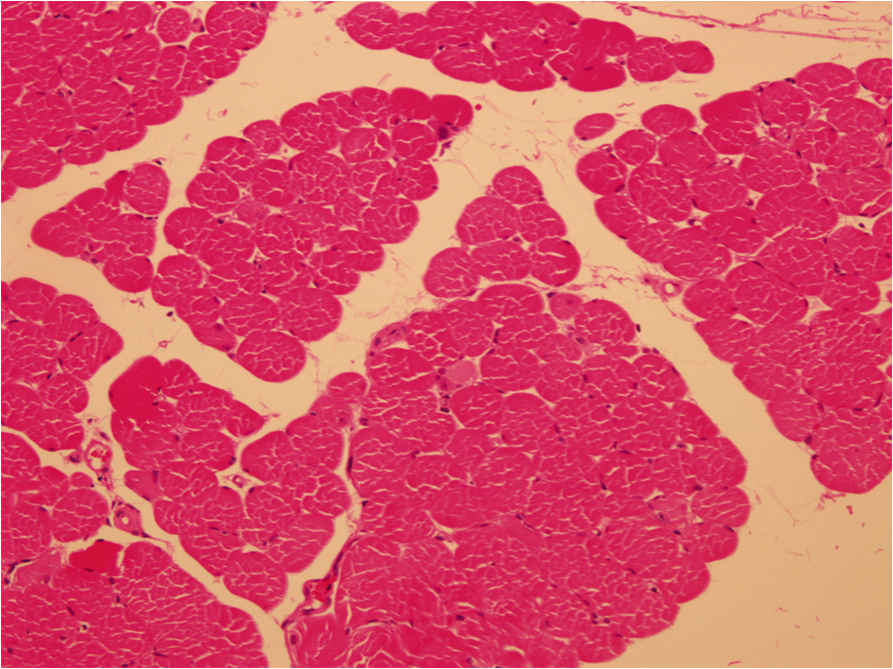
**Biopsy of the left vastus medialis muscle.** Some degenerating and regenerating fibers are visible. Hematoxylin and eosin staining, ×100.

Upon physical examination, the only notable finding was muscle weakness with a preserved gag reflex and no lateralizing or extra-pyramidal signs. Laboratory studies showed liver enzyme levels of aspartate transaminase and alanine aminotransferase to be elevated to 283 and 318, respectively. Serum levels of the muscle enzymes creatine phosphokinase (CPK), lactate dehydrogenase (LDH), myoglobin, and creatine kinase-MB (CK-MB) were markedly elevated (21311 IU/L, 3750 IU/L, 2707 ng/ml, and 198.9 ng/ml, respectively). The level of cancer antigen-125 was slightly elevated (59.5 U/ml), but other tumor markers were within the normal ranges. In addition to the physical examination and laboratory studies, we also performed a nerve conduction study and electromyography, and the patient’s muscle weakness was suggestive of a myopathy. Examination of a biopsy specimen of the left vastus medialis muscle showed findings consistent with those of primary myopathy with myofibrillar disarray, suggesting PNM (Figure 1).

**Figure 2:**
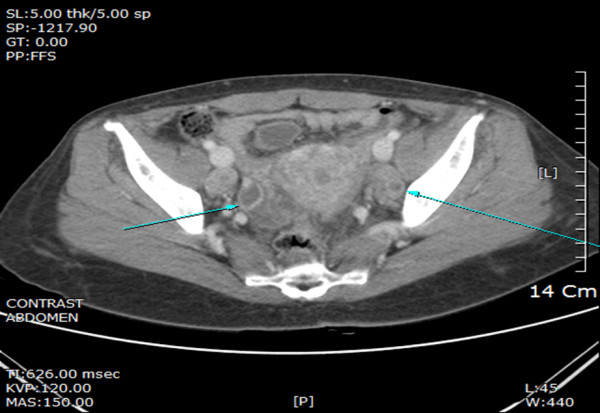
**Abdominopelvic computed tomography (CT).** Both ovaries appear normal, but the right ovary is slightly enlarged.

In addition to examining the muscles, we also sought imaging studies and located an unknown primary malignancy. Chest computed tomography (CT) showed mild interlobular septal thickening in both lungs, consistent with pulmonary edema and passive, subsegmental atelectasis in both lower lobes. Enlarged lymph nodes were visible in the right hilum, left supraclavicular, and right cardiophrenic areas, along with a small right pleural effusion. An abdominopelvic CT scan revealed scattered seeding in the entire peritoneal cavity. Both ovaries appeared normal, but the right ovary was slightly enlarged (Figure 2). Pelvic magnetic resonance imaging (MRI) showed two bilateral metastatic lymph nodes in the pelvic cavity and a 2 cm enhancing soft tissue nodule suggestive of metastasis in the left perirectal space.

Based on the imaging suggesting the presence of a primary malignancy and the likely paraneoplastic myopathy, explorative laparotomy was performed. The right ovary was visualized as a 2 cm multicystic solid mass, and the left ovary was 2.5 cm and normal in appearance. Adenocarcinoma was found during frozen tissue examination of the right ovary, and we subsequently performed staging surgery. Low anterior resection was also performed because of suspected rectal invasion of the cul-de-sac. No palpable nodules were observed on the liver surface, spleen, stomach, or appendix.

After the laparotomy, histopathological analysis of the entire specimen revealed a grade 3 ovarian serous adenocarcinoma. The right ovary measured 2 × 1.8 cm, and the left ovary measured 2.5 × 1.7 × 1.5 cm. Involvement of the right ovarian surface and fallopian tube was found, but the regional lymph nodes were spared. The resected rectum revealed direct invasion of the serous adenocarcinoma and metastases in 12 of 17 pericolic lymph nodes. Postoperatively, the patient underwent adjuvant chemotherapy with paclitaxel and carboplatin. Muscle weakness improved, and CPK levels gradually decreased.

## Conclusions

Our report describes a patient who experienced muscle weakness and myalgia as the initial presenting symptoms of a primary ovarian malignancy. In this case, there was no evidence of alternative causes of myopathy, such as other malignancies or medications. To our knowledge, this is the first report of PNM associated with EOC.

Malignancy-associated myopathy has been anecdotally reported in the past, and among these reported myopathies, idiopathic inflammatory myopathies (IIMs), mainly dermatomyositis and polymyositis, were found to be associated with cancer [5]. Specifically, the most common histological type of IIM-related cancer is adenocarcinoma [5]. According to a population-based study, dermatomyositis is more strongly associated with ovarian cancer patients than with the general population [11], but the clinical correlation between cancer and inflammatory myopathy can vary. A malignancy may occur before or in parallel with the diagnosis of inflammatory myopathy. Usually, cancer is found within three years of a myositis diagnosis, but the risk of cancer is highest at the time of diagnosis [5,11,12]. The relationship between cancer and myositis was explained by humoral immunologic mechanisms [12], and it is thought that the immune-mediated destruction of muscle may be a type of paraneoplastic manifestation of the immune system’s response to the cancer.

One paraneoplastic syndrome, PNM, is characterized by a symmetric and proximal myopathy accompanied by increased levels of serum muscle enzymes such as creatine kinase. Furthermore, nerve conduction studies and electromyography findings in PNM are suggestive of myopathy. PNM is uniquely distinguishable from other myositides such as dermatomyositis or polymyositis in three ways. First, PNM is characterized by predominant necrosis of muscle fibers with limited inflammation. Additionally, glucocorticoids are the treatment of choice in dermatomyositis, and PNM may not respond to corticosteroid therapy. Lastly, although dermatomyositis may have a clinical course independent of the cancer treatment, the prognosis of PNM depends on the underlying malignancy. Consequently, the prognosis for PNM is worse, with greater mortality [13]. Therefore, diagnosis of the underlying malignancy is critical to the management of these patients. The successful treatment of the malignancy is the mainstay of relieving the myositis symptoms.

In this study, we presented a case of ovarian serous adenocarcinoma with concomitant PNM. Although never reported until now, the possibility of EOC should be considered in unknown primary malignancies with initial complaints of muscle weakness or myalgia.

### Consent statement

Written informed consent was obtained from the patient for publication of this case report.

## Abbreviations

EOC: Epithelial ovarian cancer; PNM: Paraneoplastic necrotizing myopathy; CPK: Creatine phosphokinase; LDH: Lactate dehydrogenase; CK-MB: Creatine kinase-MB; CT: Computed tomography; MRI: Magnetic resonance imaging; IIMs: Idiopathic inflammatory myopathies.

## Competing interests

The authors declare that they have no competing interests.

## Authors’ contributions

KJM, YTO and JHH participated in the care of the patient and wrote the article. KAS participated in the care of the patient. HRH and JKL: participated in the writing of article. KJM and JHH validated content and form of the article. All authors read and approved the final manuscript.
